# Environmental Influences on the Growing Season Duration and Ripening of Diverse *Miscanthus* Germplasm Grown in Six Countries

**DOI:** 10.3389/fpls.2017.00907

**Published:** 2017-05-30

**Authors:** Christopher Nunn, Astley Francis St. John Hastings, Olena Kalinina, Mensure Özgüven, Heinrich Schüle, Ivan G. Tarakanov, Tim Van Der Weijde, Aleksander A. Anisimov, Yasir Iqbal, Andreas Kiesel, Nikolay F. Khokhlov, Jon P. McCalmont, Heike Meyer, Michal Mos, Kai-Uwe Schwarz, Luisa M. Trindade, Iris Lewandowski, John C. Clifton-Brown

**Affiliations:** ^1^Institute of Biological, Environmental and Rural Sciences, Aberystwyth UniversityAberystwyth, United Kingdom; ^2^School of Biological and Environmental Sciences, University of AberdeenAberdeen, United Kingdom; ^3^Department of Biobased Products and Energy Crops, Institute of Crop Science, University of HohenheimStuttgart, Germany; ^4^Field Crops Department, Konya Food and Agriculture UniversityKonya, Turkey; ^5^German Agrarian CentrePotash, Ukraine; ^6^Moscow Timiryazev Agricultural Academy, Russian State Agrarian UniversityMoscow, Russia; ^7^Department of Plant Breeding, Wageningen UniversityWageningen, Netherlands; ^8^SchwarzBraunschweig, Germany; ^9^Blankney EstatesBlankney, United Kingdom

**Keywords:** miscanthus, ripening, senescence, modeling, multi-location

## Abstract

The development of models to predict yield potential and quality of a Miscanthus crop must consider climatic limitations and the duration of growing season. As a biomass crop, yield and quality are impacted by the timing of plant developmental transitions such as flowering and senescence. Growth models are available for the commercially grown clone *Miscanthus x giganteus* (*Mxg*), but breeding programs have been working to expand the germplasm available, including development of interspecies hybrids. The aim of this study was to assess the performance of diverse germplasm beyond the range of environments considered suitable for a Miscanthus crop to be grown. To achieve this, six field sites were planted as part of the EU OPTIMISC project in 2012 in a longitudinal gradient from West to East: Wales—Aberystwyth, Netherlands—Wageningen, Stuttgart—Germany, Ukraine—Potash, Turkey—Adana, and Russia—Moscow. Each field trial contained three replicated plots of the same 15 Miscanthus germplasm types. Through the 2014 growing season, phenotypic traits were measured to determine the timing of developmental stages key to ripening; the tradeoff between growth (yield) and quality (biomass ash and moisture content). The hottest site (Adana) showed an accelerated growing season, with emergence, flowering and senescence occurring before the other sites. However, the highest yields were produced at Potash, where emergence was delayed by frost and the growing season was shortest. Flowering triggers varied with species and only in *Mxg* was strongly linked to accumulated thermal time. Our results show that a prolonged growing season is not essential to achieve high yields if climatic conditions are favorable and in regions where the growing season is bordered by frost, delaying harvest can improve quality of the harvested biomass.

## Introduction

Miscanthus is a rhizomatous perennial grass of Eastern Asian origin that is cultivated in the USA and Europe for its stem biomass (Clifton-Brown et al., 2001; Heaton et al., 2010). The senesced stems are currently mainly used for heat generation through combustion. *Miscanthus* shows exceptional productivity in temperate zones partly owing to its lower temperature tolerance than other C_4_ species such as *Zea mays* (maize) (Wang et al., 2008).

The seasonal pattern of growth of Miscanthus follows a cycle of: spring emergence, leaf expansion, flowering, senescence, and dormancy. The ability of *Miscanthus* to emerge early in spring has been shown to be a key determinant of final yield in the UK (Davey et al., 2016). It was projected using simulation modeling that decreasing the base temperature (T_b_) for spring emergence from 10° to 9°C would result in a 12% increase in intercepted photosynthetically active radiation (iPAR) (Davey et al., 2016). Flowering time has also been shown to influence final yield as early flowering genotypes produce lower yields than those that flower late or not at all (Clifton-Brown et al., 2001; Jensen et al., 2011). Flowering has also been linked to senescence (Wingler et al., 2008, 2010) which marks the end of biomass accumulation for that year. The timing of senescence is critically important for the remobilization of mineral nutrients and carbohydrates back to the rhizome for storage over winter and to drive shoot re-growth in spring. If this process occurs too early yield is reduced but if it occurs too late and the still-active stems are killed by a frost, the nutrients are not all remobilized and the long-term sustainability of the crop may be negatively affected (Robson et al., 2012; Purdy et al., 2014). The timing of the onset of senescence and the rate of its progression also determines the quality of the final biomass. If the timing is late or rate too slow then the crop does not dry-down (ripen) completely before harvest which results in moisture and nutrients being present in the harvested material. This has major impacts on post-harvest microbial spoilage before utilization, transport, and impairs thermal conversion efficiency (Lewandowski et al., 2003; Robson et al., 2012).

Miscanthus is generally harvested at the end of winter after senescence is complete and before emergence of new shoots (January–March). This means that the yield potential is determined by environmental conditions through the growing season combined with the environmental conditions from the end of the growing season until harvest. Temperature has been shown to control growth-rate in grass, over-riding the effects of light and circadian rhythms (Matos et al., 2014). In *Miscanthus*, cold spring temperatures limit the leaf elongation rate and cold autumn night-time temperatures can accelerate senescence and over-ride favorable warm day-time temperatures (Farrell et al., 2006; Purdy et al., 2014). A greater understanding of the effects of regional climate on plant development would help identify the optimal genotype to grow in a particular location.

Most of the research on *Miscanthus* has been carried out on a single genotype, the sterile hybrid, *M. x giganteus*. However, enormous diversity exists in the species and breeding programs are generating new elite varieties to expand the European market (Clifton-Brown et al., 2008) and the ability to predict the yield performance of diverse genotypes in a range of climatic regions would accelerate the release of new varieties tailored to specific locations.

Process based models for traits such as leaf expansion, radiation use efficiency (RUE) and the ability of the canopy to intercept light [the canopy extinction coefficient (k)] have previously been developed for *Miscanthus* (Clifton-Brown et al., 2000, 2004; Hastings et al., 2008, 2009; Davey et al., 2016). However, the genotypes used to parameterize the existing models are clonal, wild-type germplasm whereas breeding research is focused on the production of elite seed propagated varieties (Clifton-Brown et al., 2016). To address this gap in the modeling data we have built on the existing MiscanFor (Hastings et al., 2009) model that predicts biomass accumulation using all the trait models described above and including the principles of Monteith (1978). This model uses thermal time as a driver for plant development and cumulative radiation intercepted to calculate biomass accumulation. Cumulative degree days above a threshold base temperature (T_b_), determine the beginning and end of the growing season, constrained by the first autumn frost and soil moisture (Hastings et al., 2009). The cumulative degree day calculation (DDc) is a measure of the accumulation of temperature units from stem emergence to each developmental stage. It is used to derive the physical status of plant development in the model (Hastings et al., 2009) and is particularly useful for comparing *Mxg* performance at different sites as it accounts for the faster growth in warmer climates. However, the senescence and ripening processes have been poorly characterized in experiments in the past and thus the current model process descriptions of the plant dieback and biomass ripening are rather crude.

The objective of this study is the investigation of parameters used in existing *Miscanthus* models, such as MiscanFor, to extend their use to new hybrids in different climatic and soil environments by providing an improved description of the timing of key developmental stages and growth and ripening rates. This was achieved in instrumented and replicated field trials that were established at six sites in a longitudinal and latitudinal gradient across Europe planted with 15 new genotypes developed by *Miscanthus* breeding programs located in Aberystwyth (UK), Wageningen (Netherlands), and Braunschweig (Germany). Soil at each site was characterized by coring down to 1 m to determine soil texture, profile depth, and calculated plant available water. The climatic conditions and soil temperatures were monitored by on-site weather stations for 4 years from planting and phenotypic and yield measurements were taken over 2 years (Kalinina et al., 2017). Statistical analysis indicated that there was a strong interaction between genotype and environment (including soil and climatic conditions; Kalinina et al., 2017).

We hypothesized that novel germplasm that could extend the growing season duration, either by early emergence or later senescence, would lead to a greater yield without a sacrifice in biomass quality (through increased moisture content). Further to this, we anticipated matching germplasm best suited to the diverse environments of the six widely distributed trial locations.

There was large variation in the progression of ripening and the traits leading to the end of the growing season with species type and location with some genotypes not flowering at all and growth continuing in others after the initiation of flowering. In locations with significant periods of sub-zero temperatures, the time between thawing and harvest did affect the harvest quality. The yields were produced at the location with the shortest growing season from emergence to peak yield.

## Materials and methods

### Field trials

In spring 2012, a multi-location, replicated plot trial was established at six sites around Europe. The full details of the plot establishment, agronomics and yields are reported in Kalinina et al. (2017). The sites were selected to provide a wide range of climatic conditions in Turkey near Adana, in Germany near Stuttgart, in Ukraine near Potash, in the Netherlands at Wageningen, in the United Kingdom near Aberystwyth and in Russia near Moscow (Table 1). For the remainder of this paper, the sites will be referred to by the name of the nearest town; Adana, Stuttgart, Potash, Wageningen (abbreviated to “Wagen”), Aberystwyth (abbreviated to “Aber”), and Moscow. The field trials were established on arable or horticultural land except in Aberystwyth, where the trial was planted on marginal (low quality) grassland. The trial was planted as a randomized block design (generated with Genstat 14th edition) consisting of three blocks each containing a single replicate plot of each of the 15 germplasm types (Table 2). Each 5 × 5 m plot contained 49 plants, giving a resultant planted density of just under 2 plants m^−2^.

**Table 1:** Site locations for the multi-location plot trials with long-term growing season (spring to autumn equinox) mean air Temperature (°C) and Rainfall (mm).

**Location**	**Latitude**	**Longitude**	**Altitude (m)**	**Air temperature (°C)**	**Total rainfall (mm)**	**Soil plant available water (mm)**			
						**Mean**	**sd**	**Max**	**Min**
Adana	37	35	27	26.1	75	233	34	266	126
Stuttgart	48.74	8.93	463	16.4	378	144	54	268	56
Potash	48.89	30.44	237	18.5	300	260	4	262	241
Wageningen	51.59	5.39	10	15.8	376	141	17	176	95
Aberystwyth	52.43	−4.01	39	13.8	401	55	15	84	15
Moscow	55	37	140	14.8	347	202	16	251	160

*The range of soil water holding capacity (PAW, mm) across each site is shown with a mean and standard deviation for the 45 plots*.

**Table 2:** Germplasm selected for the multi-location trials.

**Genotype ID**	**Species**	**Accession details**	**Propagation method**
OPM-1	Sac	Wild Sac	*in vitro*
OPM-2	Sac	Wild Sac	*in vitro*
OPM-3	Sac	Wild Sac	*in vitro*
OPM-4	Sac	Wild Sac	*in vitro*
OPM-5	Hybrid	Wild Sin × Wild Sac	*in vitro*
OPM-6	Hybrid	Wild Sac × Wild Sin	*in vitro*
OPM-7	Hybrid	Wild Sac × Wild Sin	*in vitro*
OPM-8	Hybrid	Wild Sac × Wild Sin	*in vitro*
OPM-9	Hybrid (*Mxg*)	Wild Sac × Wild Sin	*in vitro*
OPM-10	Hybrid	Wild Sac × Wild Sin	*in vitro*
OPM-11	Sin (Goliath)	Wild Sin × open	*in vitro*
OPM-12	Sin	Wild Sin × open	seeds
OPM-13	Sin	Sin × Sin	seeds
OPM-14	Sin	Sin × Sin	seeds
OPM-15	Hybrid	Sac × Sin × open Sin (open-pollinated hybrid with dominating Sin phenotype and high morphological variability)	seeds

*Sac, M. sacchariflorus; Sin, M. sinensis; hybrid, M. sinensis × M. sacchariflorus hybrid. Common clone names added where these exist [e.g., Mxg, M. x giganteus; Sin (Goliath), M. sinensis Goliath]*.

### Plant material

Clonal genotypes and seeded types were selected from the different genetic collections of *Miscanthus* spp. in United Kingdom, The Netherlands and Germany. They were provided by Aberystwyth University, Wageningen University and SCHWARZ consulting (Kalinina et al., 2017). The 15 germplasm types included four selected genotypes of wild *M. sacchariflorus* collected from 31° to 37° latitude, five interspecies hybrids of *M. sacchariflorus* and *M. sinensis*, four *M. sinensis* seed based population hybrids (two of which were paired crosses, and two open pollinated) and two triploid standard clones: *M*. *x giganteus* [between *M. sinensis* and *M. sacchariflorus*, (Greef and Deuter, 1993)] and *M. sinensis* “Goliath” [*sinensis x sinensis*,(Purdy et al., 2013)] (Table 2).

### Weather data

Weather data was taken from a meteorological station at or within 1 km of each experimental site collecting the following data in daily time steps: daily maximum and minimum air temperature (at 2 m height), soil temperatures (at 5 cm depth), daily rainfall, daily wind run, relative humidity, and cumulative daily solar radiation. This data was recorded from the planting date to the end of the 4 year duration of the experiment. Prior long term monthly average data was available for at least 5 years at each site (Table 1). Photosynthetically active radiation was estimated as half the incident solar radiation (Jones, 1992) and potential evapotranspiration (PET) was calculated as from the Penman-Monteith (P-M) equations (Monteith, 1965).

To ensure sufficient moisture for good root establishment, during planting approximately 2 liters per plant irrigation was applied at all sites. Adana used drip irrigation to supplement rainfall during each year as required for plant health in the Mediterranean climate. Each irrigation event timing and quantity was recorded.

### Soil moisture

The soil plant available water holding capacity at each site was characterized by a comprehensive soil coring and analysis program. Soil cores were taken across the trial plots in a regular pattern using a 8.54 cm diameter cylinder corer (Eijkelkamp) driven into the ground with tractor mounted hydraulics until bed rock was reached or 1 m depth. The depth of soil was recorded and soil profile photographed. The texture along the profile was determined using the Fitzpatrick hand method (Fittzpatrick, 1992) and the stone content and bulk density measured. These physical parameters were used to calculate the wilt point and soil water holding capacity in mm using the (Campbell, 1985) method as modified by Hastings et al. (2014). The difference between the wilt point and field capacity is the plant available water in mm (PAW) (Table 1).

The drought stress through the growing year was estimated by calculating the soil moisture deficit (SMD) at each day from the date of planting. This was calculated using the daily potential evapotranspiration modified by the ratio between the daily soil water balance and the PAW using the method proposed by (Aslyng, 1965) to give an actual evapotranspiration (AET). The PAW was used as the soil water capacity which was increased by the rainfall and irrigation and decreased by the AET to calculate daily soil water balance. On days where the SMD fell below 80% of the PAW the plants were considered to be suffering from drought stress.

### Plant development measurements

During the growing season of 2014 detailed growth measurements were taken from three to five plants in the central measurement area of nine plants. The *Mx. sacchariflorus* genotypes (OPM-1 to OPM-4) have a more spreading rhizome making individual plants more difficult to distinguish. For these genotypes, all plant specific measurements used a marked area of 0.5 m^2^ centerd on the original planting location. Plant height measurements were taken regularly from emergence to the end of the year. The initial height (e_hgt) was measured from ground to the tip of the newest leaf. Emergence date was determined by a linear regression of the e_hgt measurements up to 40 cm. After the formation of the first ligule, stem height (s_hgt) was measured bi-monthly from ground to the highest ligule on the tallest stem of the plant. After canopy formation, an additional canopy height measurement was taken weekly for each plot to provide better detection of temporal changes in growth rate.

### Annual harvest yield

Harvest was performed annually in spring following the growing season between February and April depending on local climatic conditions (Table 3). The nine plants (4.59 m^2^) in the central area of each plot were cut manually with a hedge trimmer to a target cutting height of 5 cm above the soil surface. Harvested plant material was dried to constant weight at 80°C and moisture content was determined. Dry matter yield was calculated as tones of dry matter per hectare. Stem density and plant height were recorded at the end of the growing season (in October–November) on the marked plants in the middle of the plot.

**Table 3:** Date of the yield harvest at each of the six OPMTIMISC sites after the 2014 growing season.

**Location**	**Harvest date**
Adana	14/02/2015
Stuttgart	18/03/2015
Potash	23/02/2015
Wagen	04/03/2015
Aber	03/02/2015
Moscow	13/03/2015

### Serial harvests to estimate standing crop during growth

To measure biomass accumulation and leaf:stem ratios, stem samples were harvested monthly from each plot. To minimize damage to the plots, the stem samples were taken from the row between the border and central measurement area. For each plot, eight stems were harvested, two from each of four plants. The stems were selected from one side of the plot, alternating with each month so that each plant was only sampled once every 4 months. The selection was randomized using a marked stick placed through the row of plants, with the closest stem to each of the eight marks selected. Only stems with a height >60% of the canopy height were included; this excluded any newly emerging stems which would have distorted the leaf stem ratios and stem weights (Davey et al., 2016). The samples were then separated into leaf and stem material, and weighed. The leaf length and maximum leaf width were measured, as was the total leaf area where equipment was available. The leaf and stem material was oven dried to constant weight to determine the moisture content.

Serial cut dry weights were used to estimate the harvestable above ground biomass for each plot through the growing season. The ratio of the eight stem weight at each serial cut date and the eight stem weight at the final harvest was used to back calculate the standing crop biomass for each sampling date from emergence to harvest.

### Flowering

The plots were examined regularly for evidence of the transition from vegetative growth to flowering. Flowering and seed set were classified with four stages to determine the initiation of flowering, panicle emergence and the duration of anthesis; with a score given for the plot as a whole and also for the individual measurement plants to give detail on when flowering began and also how consistent this flowering was between plants within a plot. The score system recorded first emergence of a flag leaf (1), panicle emergence >1 cm (2), panicles present on >50% mature stems in a plot (3), and flowering complete on all plots (4).

### Senescence

To monitor the onset of senescence, each plot was visually assessed and assigned a score from 0 to 10 (Robson et al., 2012). This score was based on the ratio of the green to brown plant material in a plot, with a score of 0 indicating all green and a score of 10 indicating complete senescence. To ensure consistency, measurements were taken from the same direction for each plot and photographs were used to calibrate scoring between locations. This score was measured approximately fortnightly from the start of autumn until hard frost or completion of senescence.

### Data analysis

Data analysis was performed using the statistical programming software R (R Core Team, 2016). To assess the ripening of the crop, three key stages were identified: the date of peak above ground biomass, the initiation of flowering and the initiation of senescence. While all sites followed the same phenotyping protocol, there was variation in the measurement dates between sites. To synchronize measurements between locations the progression of each of the key ripening indicators (peak yield, flowering and senescence) was modeled with a curve fitting regression to predict the value for each day of the year (DOY).

Model fitting and prediction for the growth curve was performed using the base stats package in the statistical programming software R (R Core Team, 2016). The serial cut stem weight measurements for the three replicate plots for each genotype at each location were used to generate a loess curve regression analysis (span = 0.7 degree = 2). The date of the maximum value of this growth curve was used to define the date of peak yield.

Model fitting and prediction for the progression of flowering was performed using the dose response model package for R, *drc* (Ritz et al., 2015). For each genotype at each location, a two parameter log-logistic function was fitted to the flowering score (minimum = 0, maximum = 4) against day of year. This regression model was then used to predict a day of year for each flowering stage (1–3). Because of the nature of the logistic curve, this model could not be used to predict the date for end of flowering.

To summarize the progression of senescence, a similar system to that reported in Robson et al. (2012) was used. This defined two measurements; a mean senescence score and the time to reach a given threshold value. The mean score was calculated from 1st September until the harvest date. As senescence was not measured in late winter and spring, this average does not include the browning effect of frost events. To include this effect, a second summary value was included for sites which experienced a hard frost. For these sites, the senescence score was set to 100% after a frost event below −3°C. The threshold values of interest were 20, 50, and 80% senescence and the time to reach these thresholds were calculated in number of days and thermal time from emergence.

To prevent distortion of these summary values due to the effect of variation in the frequency and number of measurements between locations, a two parameter log-logistic function was fitted to the senescence scores for each genotype at each location, using the dose response model package for R, *drc* (Ritz et al., 2015).

To compare the progression of flowering and senescence to biomass accumulation, the scores were normalized. The flowering score (0–4) was divided by four and the senescence score (0–10) was divided by ten. The estimated dry matter for each plot was divided by the equivalent dry matter at harvest giving a value for the proportion of the final harvest weight standing in the field through the year.

To compare plant development between sites thermal time was calculated based on the air temperature measurements of the accumulation of degree days from emergence to each of the development stages. Many studies (Snyder et al., 1999; Cesaraccio et al., 2001; Hastings et al., 2009) have found a T_b_ of 0°C suitable, and this was used to report thermal time values in this paper.

Statistical comparison of the species and location main effects have been were performed using the HSD (honest significant difference) function of the agricolae package for R (De Mendiburu, 2016)

## Results

### Weather data

This study focussed on the third year of growth (2014), when at most locations the plants are physiologically mature and canopy closure occurs in most genotypes at most sites within the growing season. While all locations showed some variation from the long term average, especially in rainfall; of interest to this study was the response of the crop to the six different growing conditions in 2014.

In 2014, the average “growing season” temperature gradient, from high to low, followed an East-West trend. The coldest winter temperatures (mean minimum air temperature) showed a West-East trend with the exception of Adana, which is over 11° further south than Stuttgart, the next most southerly of the six test sites. This also created a gradient of annual temperature range, where the difference between summer and winter increased from West to East, as the site climates became more continental.

**Figure 1:**
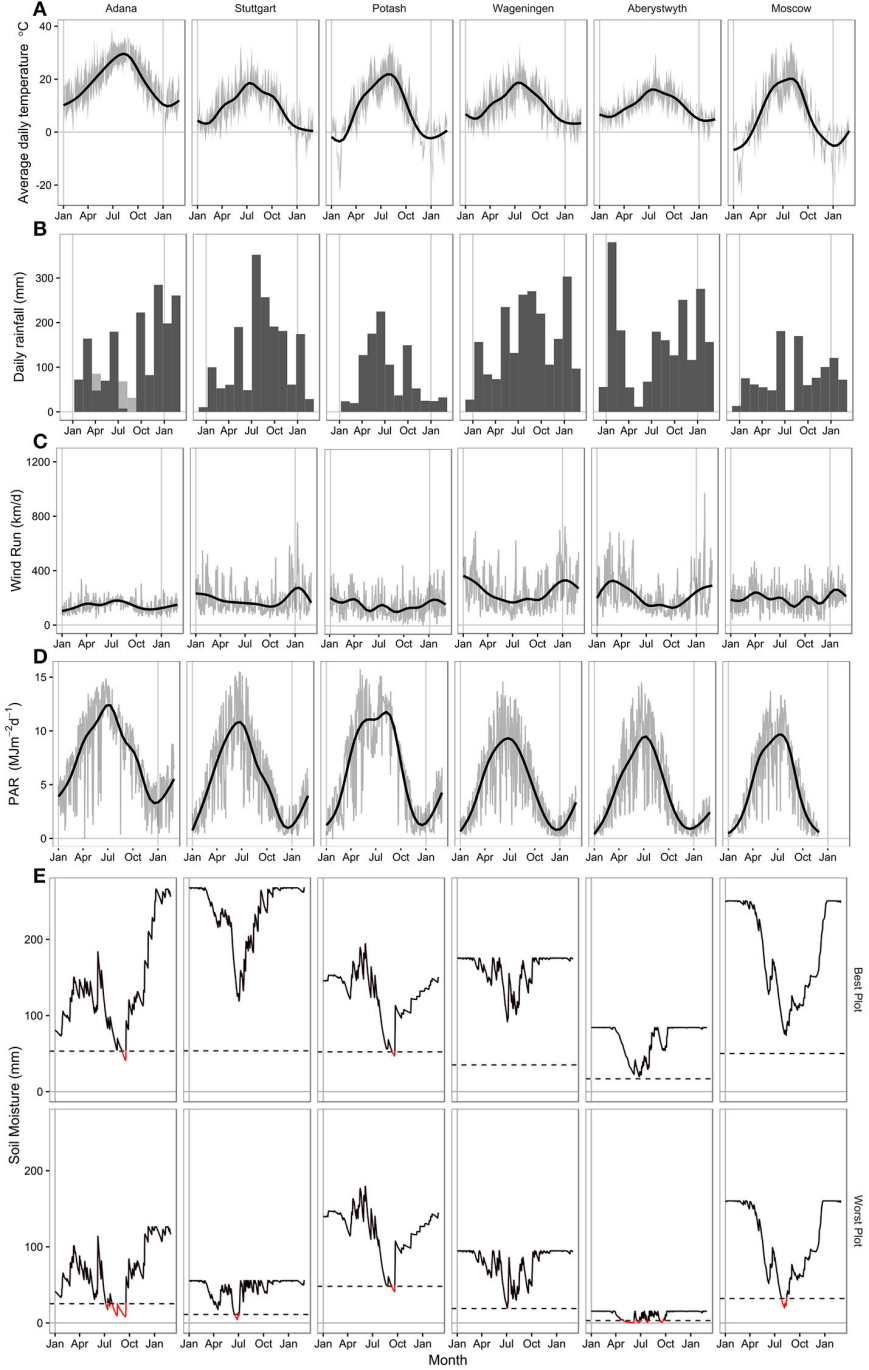
Climate data at each location for the 2014 growing season from 1st January 2014 to 1st April 2015. **(A)** Smoothed daily mean air temperature (2 m height) with a gray ribbon showing daily maximum and minimum air temperatures. **(B)** Total monthly rainfall (mm) with irrigation shown with hatched bars at Adana. **(C)** Smoothed daily wind run (km/d) with gray line showing daily values. **(D)** Smoothed photosynthetically active radiation (MJm^−2^d^−1^) with gray line showing daily values. **(E)** Modeled daily soil moisture (mm) at the plot with the best and worst water holding capacity (PAW) at each location. The dashed line shows 20% PAW, below which the crop is considered to be water stressed.

For five out of the six sites, growing season temperatures in 2014 were similar to the long term averages. In Moscow, summer temperatures were significantly higher than long term averages (Figure 1A, Table 1). Overwinter temperatures fell below freezing at all locations; however, only at Potash and Moscow was there extended periods with a mean air temperature below 0°C. Minimum air temperatures at Adana did not fall below –2°C and at Aberystwyth did not fall below −3°C until mid-January.

Rainfall varied in 2014 compared to the long term average at all locations in this trial (Figure 1B, Table 1). Adana had a drier winter and wetter summer than usual, with high rainfall in June 2014. Stuttgart also had a much wetter summer than normal, with heavy rainfall in July and August. Potash had a drier winter and much wetter April and May than the average. Total growing season rainfall at Wageningen was fairly normal, although May, July and August were wetter than normal. Spring through to summer was dry at both Aberystwyth and Moscow with lower than average growing season rainfall amounts in both sites. From April to October rainfall exceeded 50 mm every month at only two sites; Stuttgart and Wageningen (Figure 1B). At all other sites rainfall dropped below this value for at least 1 month leading to some water deficits at different times during the growing season. In Adana and Moscow, rainfall dropped to <10 mm in late summer, whereas in Aberystwyth rainfall dropped below 10 mm near the beginning of the growing season in May.

Incident photosynthetically active radiation (PAR, MJ m-2 d-1), which was estimated from 50% of global radiation at some sites, was highest in Adana throughout the year (Figure 1D). In Potash a drop in PAR was observed in June that corresponded with a period of high rainfall.

Most locations suffered some soil water deficits (mm) in the 2014 growing season (Figure 1E) which approached the plant available water (PAW, mm) holding capacity of the soil profile. In Adana, despite irrigation, calculated soil moisture deficits exceeded 80% of the plant available water (PAW) from June to October. In Stuttgart there was sufficient summer rainfall to ensure that water was non-limiting throughout most of the growing season in plots with soil depths >60 cm. Across the Stuttgart field site, soil depth varied markedly (from 30 to 100 cm) resulting a large variation in calculated soil PAW. Soil moisture deficit below 80% of the PAW occurred in June and July in the shallower plots.

At Potash, warm growing season temperatures and high atmospheric vapor pressure deficits caused by lower summer relative humidities than at the other sites led to large soil moisture deficits. However, as the soil is a high quality chernozem, which is highly water retentive, water supply in Potash was unlimited for most of the 2014 growing season and soil moisture deficits only exceeded the estimated PAW in September and October (Figure 1E). At Wageningen, where there was good distribution of rainfall throughout the growing season, the sandy soil textures meant that we predict there was mild water deficit in June in some of the poorer plots though these don't appear to have produced detectable changes in growth rate (Figure 1E). The site in Aberystwyth has the poorest soils of all the six locations. Soils varied from 30 to 60 cm depth across the site, with PAW's from 16 to 84 (mm). The spring of 2014 was dry; with total rainfall between March and May of 127 mm. SMD exceeded 80% of the PAW from June to October, severely restricting the water available for growth. In comparison to Aberystwyth, Moscow has generally good quality and deep soils. However, during a dry and hot period in August 2014, SMD fell rapidly and exceeded 80% PAW in the shallowest plots significantly reducing late summer season growth rates.

### Plant development

#### Emergence

**Figure 2:**
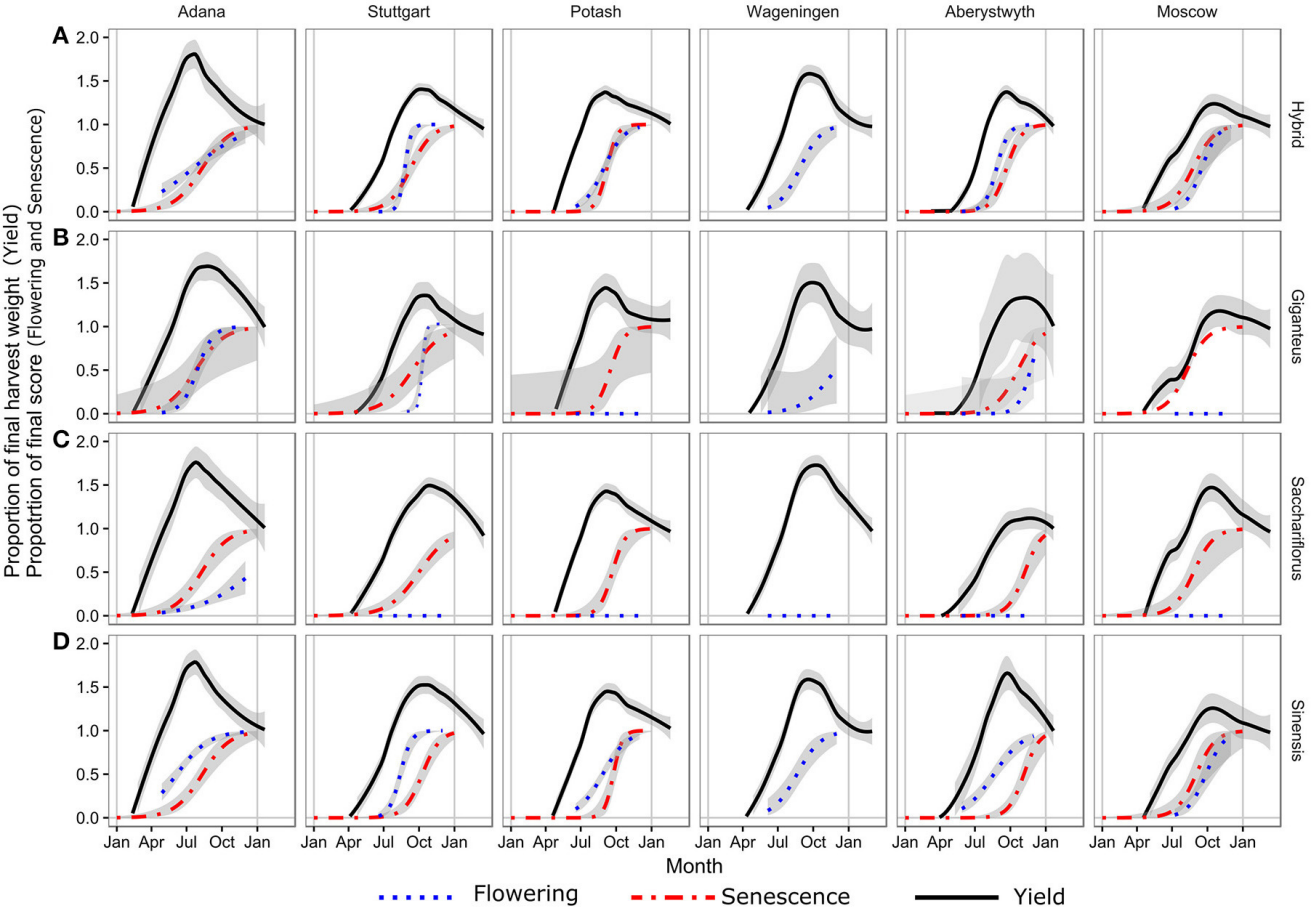
Regression curves for the three ripening traits. Fitted curve to the serial cut yield estimates normalized to the final harvest in spring as a black line. Fitted curve for senescence scored at plot level based on weekly to monthly measurements as red line. Fitted curve to the flowering score as blue line. The gray shading is 1 standard error on the curve fits (n varies). Flowering score 0–4 (5 steps) has been normalized to 0–1.

In all species, spring emergence date, flowering time, the date of peak biomass and senescence all occurred earliest at Adana (Figure 2, Table 4). Emergence at Adana occurred in early February, at least 40 days before any other location and before the predicted date used by MISCANMOD. Emergence at Aberystwyth, Stuttgart and Wageningen occurred around the spring equinox (day 78) as expected (Table 4A); although some of the hybrid and *Mx. sinensis* genotypes emerged earlier. Emergence dates at Potash and Moscow were significantly later than in all other sites (day 102 and 106 respectively). Generally the thermal time was similar between locations at between 350 and 450 DD_0_. The thermal time to emergence was highest at Wageningen (609DD_0_ for *Mxg*) and lowest at Moscow (191DD_0_ for hybrids). Thermal time to emergence was much lower at Moscow despite the late emergence date.

**Table 4:** Ripening traits in 2014 growing season by location and species group (hybrid, *M. sinensis* × *M. sacchariflorus* hybrid; *Mxg, M. x giganteus*; *Sac, M. sacchariflorus*; *Sin, M. sinensis*).

**A**	**Day of year—emergence**				**HSD group**	**Thermal time—emergence**				**HSD group**	**Daylength—emergence**				**HSD group**
**Location**	**Hybrid**	***Mxg***	***sac***	***sin***		**Hybrid**	***Mxg***	***Sac***	***Sin***		**Hybrid**	***Mxg***	***Sac***	***Sin***	
Adana	36	38	36	36	e	450	460	450	450	a	11	11	11	11	e
Stuttgart	77	81	81	76	d	350	393	393	339	b	12	12	12	12	d
Potash	104	103	104	97	b	359	350	359	303	c	14	14	14	13	b
Wagen	84	92	86	83	cd	530	609	528	514	c	13	13	13	12	cd
Aber	75	82	83	83	c	464	520	525	525	a	12	12	12	12	c
Moscow	101	104	108	106	a	191	211	246	230	c	14	14	14	14	a
HSD group	b	a	a	b		a	a	a	a		b	a	a	ab	
**B**	**Day of year—Flowering score** = **1**				**HSD group**	**Days from emergence—Fscore** = **1**				**HSD group**	**Thermal Time—Fscore** = **1**				**HSD group**
**Location**	**Hybrid**	***Mxg***	***sac***	***sin***		**Hybrid**	***Mxg***	***Sac***	***Sin***		**Hybrid**	***Mxg***	***Sac***	***Sin***	
Adana	126	182	171	121	d	90	144	134	85	d	1507	2813	2508	1396	c
Stuttgart	231	283		210	b	154	202		133	a	2159	2897		1787	a
Potash	216			205	c	112			108	c	2000			1718	ab
Wagen	211	268		198	c	127	176		114	b	1583	2641		1431	bc
Aber	220	300		185	c	145	218		102	b	1770	2816		1159	d
Moscow	240			243	a	139			137	c	2376			2380	bc
HSD group	b	a	c	c		b	a	b	c		b	a	a	c	
**C**	**Day of year—Senescence** = **5**				**HSD group**	**Days from emergence—Senescence** = **5**				**HSD group**	**Thermal time—Senescence** = **5**				**HSD group**
**Location**	**Hybrid**	***Mxg***	***Sac***	***Sin***		**Hybrid**	***Mxg***	***Sac***	***Sin***		**Hybrid**	***Mxg***	***Sac***	***Sin***	
Adana	222	201	219	222	d	184	162	181	186	c	3,985	3365	3891	4005	a
Stuttgart	256	257	277	287	b	175	173	191	207	b	2,514	2506	2781	2969	b
Potash	252	264	268	269	c	147	160	163	171	d	2,710	2925	2949	2930	b
Wagen	–	–	–	–	–	–	–	–	–	–	–	–	–	–	–
Aber	265	297	316	313	a	190	215	233	230	a	2,457	2809	3006	2968	c
Moscow	239	220	242	250	d	138	116	134	144	e	2,327	1991	2325	2459	d
HSD group	c	bc	b	a		b	b	b	a		C	c	b	a	
**D**	**Day of year—Peak**				**HSD group**	**Days from emergence—Peak**				**HSD group**	**Thermal time—Peak**				**HSD group**
**Location**	**hybrid**	***Mxg***	***Sac***	***Sin***		**hybrid**	***Mxg***	***Sac***	***Sin***		**hybrid**	***Mxg***	***Sac***	***Sin***	
Adana	180	236	223	190	e	143	197	186	153	d	2,461	4,282	3,877	2,697	a
Stuttgart	282	285	310	303	a	201	201	225	223	a	2,910	2,667	3,112	2,957	a
Potash	246	244	245	254	d	141	140	140	156	e	2,428	2,676	2,588	2,544	c
Wagen	269	268	277	263	c	187	202	205	186	c	2,326	2,677	2,559	2,562	b
Aber	264	263	280	261	b	189	181	197	178	b	2,491	2,495	2,823	2,706	cd
Moscow	291	291	291	291	c	190	187	183	185	de	2,740	2,764	2,670	2,665	d
HSD group	b	a	a	b		b	a	a	b		C	A	B	Bc	
**E**	**Peak yield (t ha**^−1^**)**				**HSD group**	**Harvest yield (t ha**^−1^**)**				**HSD group**	**Percentage biomass loss**				**HSD group**
**Location**	**Hybrid**	***Mxg***	***Sac***	***Sin***		**Hybrid**	***Mxg***	***Sac***	***Sin***		**Hybrid**	***Mxg***	***Sac***	***Sin***	
Adana	16.61	22.03	10.68	24.14	a	7.55	13	5.6	11.43	c	55	41	48	53	a
Stuttgart	19.9	18.52	16.35	16.46	a	14.11	13.55	10.49	10.38	b	29	27	36	37	bc
Potash	21.6	24.35	21.4	16.74	a	15.51	16.75	15.19	11.4	a	28	31	29	32	bc
Wagen	17.75	22.82	14.84	15.97	a	11.26	14.34	8.58	10.1	bc	37	37	42	37	b
Aber	12.17	12.15	5.22	6.8	b	8.61	8.33	3.52	3.18	d	29	31	33	53	b
Moscow	10.63	9.51	6.65	7.77	b	8.11	7.82	4.23	5.66	d	24	18	36	27	c
HSD group	a	a	b	ab		a	a	b	b		a	a	a	a	

*Pairwise comparisons use the TUKEY HSD test to assign grouping. **(A)** Timing for modeled emergence day of year, with the thermal time (Tb = 0) and daylength. **(B)** Modeled start of flowering (Fscore = 1) showing day of year (DOY), number of days from emergence and thermal time after emergence. **(C)** Timing to reach 50% senescence (Sscore = 5) showing DOY, days after emergence and thermal time after emergence. **(D)** Timing to reach peak biomass as DOY, days after emergence and thermal time from emergence. Peak yield date in Moscow is cut off where a < −3oC frost occurs on day 291. **(E)** Modeled peak yield, harvestable yield in spring and% overwinter loss*.

#### Flowering

All hybrid genotypes flowered at all sites, with one unexpected exception; OPM-8 did not flower at Adana, although flowering was earliest at this site for all other genotypes. *Mxg* flowered in four out of the six sites. Three of the four “pure” *Mx. sacchariflorus* genotypes did not flower at any location. The only *Mx. sacchariflorus* which flowered was OPM-4 at the hottest site (Adana). All *Mx. sinensis* germplasm types flowered at all locations (Table 4B), but not all flowering plants completed flowering before the end of the growing season.

At all sites except Moscow, the *Mx. sinensis* types flowered earliest, ranging from DOY 121–185, followed by the hybrids (DOY 126–220) (Table 4B). At Moscow the hybrids and *Mx. sinensis* genotypes flowered at almost the same time (DOY 240 and DOY 243), respectively. At all the four sites where *Mxg* flowered; Adana, Stuttgart, Wageningen and Aberystwyth, it was the last species to do so (Table 4B).

In the novel hybrids, the daylength at the initiation of flowering ranged from 14 to 16 h. *Mxg* started flowering over a much greater range of photoperiods than the novel hybrids, with flowering occurring in mid-summer in Adana at 15 h, and in late autumn in Aberystwyth at 10 h (Table 4B). The *Mx. sinensis* species generally flowered at the same photoperiod or slightly longer than the novel hybrids. The greatest difference was at Aberystwyth where the *Mx. sinensis* flowered in mid-summer, when the photoperiod was 17 h and the new hybrids flowered later, when photoperiod was 15 h.

Flowering occurred much earlier in the year at Adana than any of the other locations, on average 88–98 days earlier for the hybrid and *Mx. sinensis* genotypes and 102 days earlier for *Mxg*. Using the early emergence date at Adana to calculate a number of days to flowering by subtracting the day of emergence from the day of flowering, this difference was reduced to 33 days for *Mx. sinensis*, 44 days for the novel hybrids and 54 days for *Mxg* (Table 4).

#### Senescence

The DOY to reach 50% senescence varied between locations, with the mean values ranging between DOY 201 for *Mxg* at Adana compared to 297 at Aberystwyth (Table 4C). The earliest site to reach 50% senescence for all species was Adana and the location that showed the latest senescence was Aberystwyth. The novel hybrids generally senesced earlier than the other species but during a period of large soil moisture deficit in Adana and Moscow, *Mxg* senesced earlier than all other genotypes (Table 4C). The novel hybrids showed the least variation in senescence date between sites, with the minimum and maximum DOY for 50% senescence being 222 (Adana) and 265 (Aber), respectively, giving a range of 43 days. For *Mx. sinensis* and *Mx. sacchariflorus* there was >90 days variation in senescence time within the groups and between the sites (Table 4C).

When the growing season length was adjusted to factor in emergence date, Aberystwyth remained the site with the latest senescence for all species, but although Adana had the earliest senescence, Moscow was found to have the shortest growing season measured in terms of days between emergence to senescence in all species (Table 4).

The thermal time taken to reach 50% senescence also showed large variation between sites. The hybrids showed the greatest variability, with 50% senescence at Adana corresponding to thermal time of 3,985 DD compared to 2,327 DD at Moscow, a range of 1,658 DD.

#### Growing season length

As was observed for the senescence scores, across all genotypes and sites, peak biomass was attained earliest in Adana and latest in Aberystwyth (Table 4D). Peak biomass was reached earliest in the hybrid genotypes, followed by the *Mx. sinensis* group, *Mxg* and the *Mx. sacchariflorus* group. In contrast to the senescence scores, the greatest difference between sites was observed in the novel interspecies hybrids with a range of 111 days between the earliest and latest to reach peak biomass in Adana (DOY 180) and Moscow (DOY 291). The length of the effective growing season to peak biomass varied significantly between locations (Table 4D) as expected. The longest growing season at 200–220 days was at Stuttgart where emergence occurred at day 80 and growth was not cut short by frost. The shortest growing seasons were at Potash and Adana; however the timing of growing season dates were very different with an early emergence and very early end of growth at Adana compared to a late emergence and early end of growth at Potash. These were the sites with the highest cumulative degree days. The completion of the growing season at an earlier DOY at Adana corresponds to the early flowering of the hybrids and *Mx. sinensis*, which shortened the growing season compared to the later flowering Mx. sacchariflorus genotypes and *Mxg* (Table 4).

The shortest growing season from emergence to 50% senescence was at Moscow, where emergence was delayed and senescence was accelerated due to drought. The shortest growing season duration from emergence to maximum yield was at Potash except in the *Mx. sinensis* types where the growing season was slightly shorter at Adana (Table 4D).

At the Moscow site, the growing season to 50% senescence is very short but the fitted curve from the serial cuts indicate that the biomass was still increasing at the final serial cut. However, a hard frost at day 291 would have killed any plant above ground and ended any further growth and active senescence. This gives a growing season length of 163–166 days, which is comparable to the number of days from emergence to 50% senescence at the other locations (147–236).

The longest growing seasons were generally at Stuttgart, whereas Aberystwyth had the longest duration to reach 50% senescence (Table 4C). This result demonstrates that there is no direct relationship between reaching a score of 50% senescence and peak biomass. *Mxg* and *Mx. sacchariflorus* genotypes had continued growing longer than the novel hybrids and the *Mx. sinensis* types but generally reached 50% senescence before the *Mx. sinensis*.

In the hybrids the timing of flowering and senescence was closely linked, whereas in *Mxg*, senescence tended to precede flowering (Figures 2A,B). This was in contrast to *Mx. sinensis* where the opposite was observed and flowering preceded senescence (Figure 2D). Comparing the flowering and senescence scores to the date of peak yield indicated that the biomass accumulation had peaked for all genotypes by the date the senescence score reached 0.5.

The flowering score at peak biomass was more variable, depending on species type, with the hybrid and *Mx. sinensis* genotypes in mid flowering (score of ~3) at the peak yield and just starting (score of ~1) for *Mxg* (Figure 2).

#### Yield

The highest peak yielding group on average was *Mxg* with a maximum of 24.35 t ha^−1^ at Potash (Table 4E), but the hybrids performed best at Stuttgart, Aberystwyth, and Moscow with a maximum yield of 19.9 t ha^−1^ at Stuttgart. The *Mx. sacchariflorus* genotypes were the poorest yielding at all sites except Potash where the *Mx. sinensis* species were lower yielding. Yields for all species were highest at Potash and lowest at Aberystwyth and Moscow. This demonstrates that yield was not compromised by a short growing season duration as Potash had the shortest growing season but the highest yields and the opposite was true of Aberystwyth. There was generally a positive correlation between peak and harvestable yield with the order largely remaining the same; *Mxg* was the highest yielding species and Potash the highest yielding site. Overwinter losses ranged on average between 30 and 40% for the four species. The hybrids had the greatest range between sites with 55% loss at Adana compared to 24% loss at Moscow. In the *Mx. sinensis* genotypes more than 50% of biomass was lost over winter at two sites, Adana and Aberystwyth. The site that generally experienced the greatest over-winter decline for all species was Adana and losses were lowest at Moscow.

#### Moisture content

**Figure 3:**
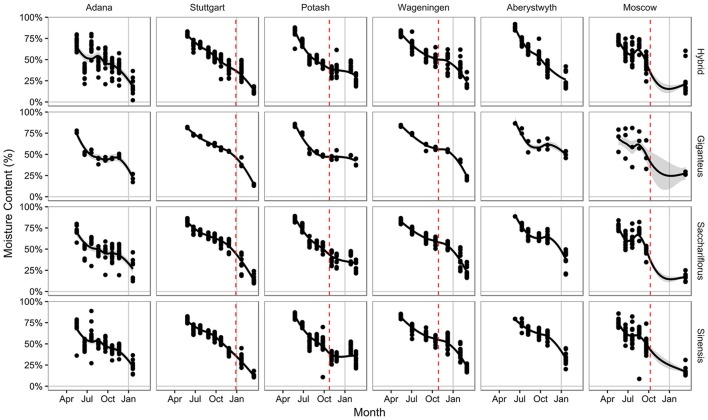
Moisture content of harvested serial cut material by location and species groups. Data points show the measured moisture content for each plot. Gray area shows one standard error from fitted curve. Red dashed line indicates the first −3°C frost of winter

The moisture content of the above ground plant material reduced through the year, starting at around 80% in all genotypes at all locations (Figure 3). In general, the pattern of change showed a decrease through the growing season, as the material changed from young green shoots to full stems. As the plant actively senesced, this decrease in moisture content continued, although the rate gradually decreased, with the moisture content stabilizing at between 40 and 50% at most locations through autumn. The greater spread of data observed at Adana and Moscow may correspond to periods of low rainfall during the growing period resulting in accelerated drying (Figures 1, 3). At the end of the year moisture contents tended to have remained higher at Potash and Aberystwyth with a maximum ~50% moisture in *Mxg* at both sites. The driest site was Stuttgart at which every genotype contained <25% water by January (Figure 3). Over winter, the rate of drying tended to increase again, due to the passive senescence effects of the environment however, this trend was not observed for germplasm grown at Potash which generally showed a plateau in drying between the first frost to −3°C and spring harvest (Figure 3). Although climatic conditions between Potash and Moscow were quite similar (Figure 1) this same trend was not observed at Moscow where all genotypes had dried down to ≤25% by the spring harvest.

**Figure 4:**
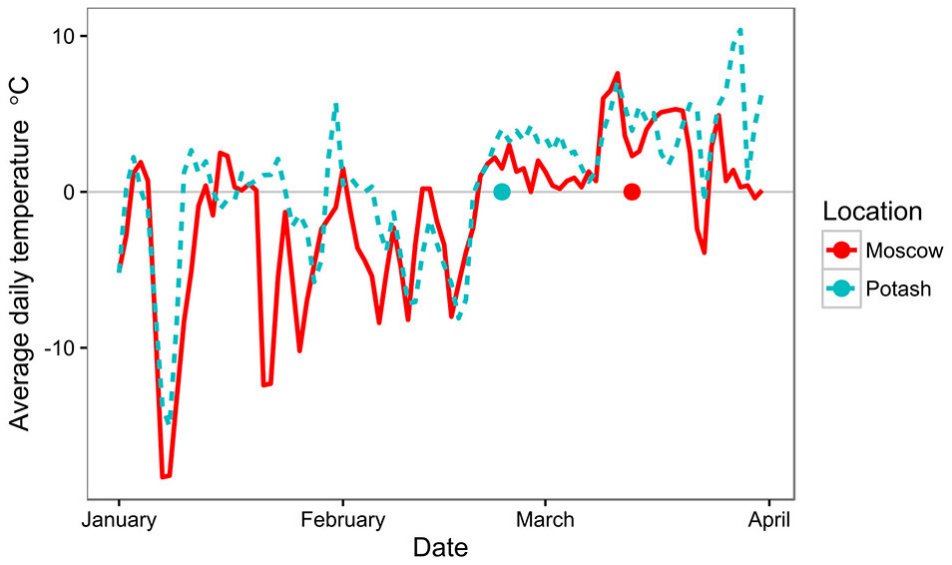
Average daily temperature at Moscow and Potash from 1st January 2015 to 1st April 2015. Harvest date at each location is indicated by a dot on the x-axis.

To further investigate why quality, in terms of drying, was compromised at Potash compared to Moscow, the climatic conditions around the final harvest were investigated (Figure 4). At both sites, the average daily temperature before 20 February 2015 was frequently sub-zero but after this date the averages were consistently 0–10°C until the end of March with very few deviations (Figure 4). However, the harvest at Potash took place within the first week of this spring thaw (23 February 2015), whereas at Moscow it was carried out nearly 3 weeks later (13 March 2015).

## Discussion

### Emergence and plant growth

The MiscanFor model for *Mxg* uses the date of the spring equinox, where the daylength exceeds 12 h at all latitudes, as a trigger for emergence date (Hastings et al., 2009). In this study, the wide variation in environmental conditions, especially over winter, provided useful new data on the triggers of emergence. At two of the trial sites (Adana and Aberystwyth) there was a lack of significant over-winter frost and at both of these locations emergence occurred before the date defined by Hastings et al. (2009). However, emergence occurred remarkably close to this date at Stuttgart, Wageningen and Aberystwyth, in all species groups. While thermal time may provide an indicator of the speed of development of new shoots, it is not a good indicator for the date of emergence as it does not easily account for frost damage. In this study, as emergence date was calculated from a regression of height measurements, any shoots killed by a frost event would not be recorded, which accounts for the delayed emergence date at Potash and Moscow.

Miscanthus is reportedly a facultative short day flowering species, though it will flower if given enough heat, even without shortening days (Jensen et al., 2013). Of the *sacchariflorus* genotypes, only OPM-4 flowered and this was only at Adana; the hottest site. This supports the findings of Jensen et al. (2013). However, the lack of flowering in the other sacchariflorus genotypes was probably due to insufficient water triggering accelerated rate of senescence. At Adana, senescence had generally passed 50% by early August which meant that when the shortening photoperiods should have induced flowering, the plants had already senesced.

The lack of correlation between thermal time and flowering date across the six sites for the hybrid and *Mx. sinensis* genotypes is consistent with previous reports and indicates that thermal time cannot be the dominant trigger for flowering (Jensen et al., 2011). However, an additional possibility is that a thermal time trigger is present for flowering, but that there is a lag involved before flowering is measurable on the plant. This would be extremely difficult to determine as the length of this lag could be variable, based on temperature, genotype, photoperiod and water deficit (Jensen et al., 2011). In several genotypes at several sites, flowering was triggered but did not complete before winter, demonstrating that the conditions experienced in the period from flag leaf to anthesis are also critical for determining whether the plant completes its life-cycle or not. This requirement adds another level of complexity to the development of models to predict flowering time.

*Mxg* does seem to require a minimum thermal time to flower; and also a minimum number of calendar days as in Moscow and Potash, *Mxg* does not flower, even though there are sufficient thermal units (>2,800 DD_0_). The growing seasons are cut short at both of these locations by frost, indicating that *Mxg* does require a minimum number of days to develop before flowering. The daylength does not seem to determine flowering at all.

In *sinensis*, flowering generally occurred earlier in the west and latest in the east, with the exception of Adana, where flowering occurred at the lowest thermal time. This latter finding is in contrast to previous reports in which flowering at a hot, southern site in Portugal occurred at a higher thermal time than the more northern sites (Clifton-Brown et al., 2001). However, the long-term average temperatures at Adana were 7°C warmer than those reported in Portugal and correspondingly flowering in *sinensis* still occurred in summer in Portugal (Clifton-Brown et al., 2001), whereas flowering was triggered in spring at Adana.

The effect of water deficit on flowering in *miscanthus* is not understood in detail and this paper has not been able to improve this. At Adana, where there were significant water deficits, flowering occurred early in the year, and at a low thermal time. However, flowering was delayed at Moscow, where there were also water deficits in the mid-summer. The other location where drought stress was significant was at Aberystwyth, and here the flowering of the *sinensis* genotypes was earlier than expected, with a very low thermal time from emergence. This would indicate that drought stress can accelerate the onset of flowering in *sinensis*, but there is not sufficient data to indicate that the hybrids follow this trend, especially as the next lowest thermal time for hybrid flowering was at Wageningen, where there was very little water deficit.

Flowering was expected to be an important trait for ripening and to mark the end of the growing season (Robson et al., 2012). However, for the hybrid and *sinensis* germplasm used in this trial, there was a period of continued growth and biomass gain after flowering. Also, genotypes which did not flower, such as the *sacchariflorus* types, did stop growing and senesced before winter.

### Senescence and the end of growth

The key definition of ripening in a biomass crop is when the rate of biomass accumulation stops, which in this paper has been termed the peak yield date. In *miscanthus*, especially when used for combustion, the end of growth also marks the beginning of an improvement in quality (Lewandowski and Heinz, 2003; Iqbal et al., 2017). However, the flowering types (hybrid and sin) lost the highest proportion of peak biomass at Adana, where flowering was early in the year and so there was an extended senescence (active and passive) period before harvest.

As with flowering, the rate of senescence was distinctive between genotypes and locations. Due to drought stress effects and general browning of leaves below the canopy, it is more difficult to determine the start of senescence. It could be reasoned that the absence of flowering in Sac and *Mxg*, at some sites, may be the cause of the extended growing season in these genotypes but in *Mxg* the longest growing season was observed at Stuttgart and Wageningen (~200 days) where flowering was observed, whereas the growing season was ~60 days shorter at Potash where flowering was not observed.

While there is a period of drought stress at Moscow which leads to a browning of the crop, growth continued in September, after rainfall and then continued until halted by frost. Clifton-Brown et al. (2002) found that with a stay green variety, photosynthesis restarted after water stress was relieved which matches the results in Moscow.

The growing season at Moscow did not start until the final frost (day 100) and flowering and senescence had not completed when growth was cut short by autumn frosts (day 291). It does not appear that a shortened growing season led to reduced yields, as the shortest growing season was at Potash, where the highest yields were recorded, and at Adana, which had the highest *sinensis* yields. The *sinensis* harvest would have been higher at Adana, however the extended period between peak yield and harvest led to large yield loses.

These results demonstrate that the relationship between flowering time, growing season duration and yield is not as deterministic as previously thought. It has previously been reported that an extended canopy duration, from earlier emergence in spring rather than delayed senescence, was critical to achieving high yields at Aberystwyth (Davey et al., 2016). However, our findings have shown the opposite; that Potash, with the shortest growing season, achieved the highest yields and Aberystwyth with the one of the longest growing seasons had the lowest yields. This indicates that the ideotype required to produce optimum yields in a temperate oceanic climate such as Wales is different to that required in a temperate continental climate like Ukraine. In Wales, where summer conditions remain comparatively cool with low PAR, a plant must capitalize on the long spring-summer daylength to achieve high biomass. However, in Ukraine where summer temperatures and PAR are high, the rate of growth is more rapid and the importance of growing season duration is minimized. These are important considerations when considering both a location specific and more generalized breeding strategy.

Generally, the pattern of change of moisture content was the same for all genotypes at a specific location, indicating that environmental conditions had a greater influence than plant morphology and senescence. This study did not find a strong link between early senescence and low moisture at harvest, except at Aberystwyth, which is the same location as a previous study (Robson et al., 2012) which found a strong link. This is a temperate oceanic environment, with mild winters and rare frosts. These findings indicate that the hardness and duration of winter frost has an effect on the above ground material, with extended periods of below freezing temperatures preventing the plants from drying out.

In future studies we will pay closer attention to the dynamics of moisture content of the crop by increasing the frequency of measurements over from monthly to bi-monthly to get smoother fits. In Moscow the snow cover detracts from the practicality of frequent sampling, but had it been done, we would have better understood the ripening profiles of different genotypes in another extreme environment which would have added to our knowledge of genotype × environment responses.

### Multi-location trials

The establishment and measurement of six trials in very different environments did pose some problems. Some measurements—such as greenness and canopy height—were based on a subjective judgment by the onsite operator; additionally even the most objective measurements such as stem height and weight could be affected by location specific practices and conditions. For example, the fresh weight of the stems collected at each serial cut will potentially by affected by the time from cut to weighing; and the environmental conditions at the time. Even the shape of the plots and space between the measurement plants can be affected by the path taken by the operator. These differences were anticipated as much as possible and prescribed in the measurement protocols. Additionally, regular meetings and in field training along with videos of measurements being taken provided examples for comparison; as did pictures of the progression of the different sites for comparison of the more subjective measurements.

## Conclusions

In this paper, we have characterized the timing of emergence from the overwintering rhizome, rate of canopy development and in season growth, flowering and senescence time, and overwinter ripening prior to harvest for 13 wild and hybrid germplasm types alongside the standard genotypes Mxg and *M. sinensis* “Goliath.”

While there was a wide “within and between site” variation in growth traits, there were several germplasm types that were generic high performers in most of the environments tested. In general, the highest yielding types were the interspecies hybrids including Mxg.

Biomass quality as expressed by moisture content at harvest was not determined by senescence and was more affected by the overwintering conditions and the time period between complete senescence and harvest. In locations with extended freezing temperatures a delay between thawing and harvest may be necessary to improve biomass quality.

Water balance calculations performed for all sites for the third growing season after planting showed the germplasm was exposed to a wide range of “in growing season” partial and severe water deficits. *M. sinensis* types were more drought resilient than the interspecies hybrids, but as these flowered earlier, they were often lower yielding. The complex germplasm responses to these dynamic water deficits can partly explain the lack of simple correlations in thermal time and photoperiod with flowering and senescence time. More work is needed to unravel these complex processes using side by side irrigated and rainfed trials with a smaller set of key germplasm types.

In temperate climates, such as the UK, strong correlations between growing season length and yield have been found in many diverse *Miscanthus* genotypes. Consequently, early leaf emergence in spring time and late flowering / senescence have long been target traits for breeders attempting to increase biomass yield. In this “EU-OPTIMISC” multi-location field experiment, which included two *Miscanthus* trial locations in strong continental climates (Moscow and Potash), simple correlations with these traits and yield were not detected. Our results indicate that, for these strong continental climates with short sharp growing seasons, breeding selections should focus high net photosynthetic rates and water use efficiency rather than the triggers and brakes determining the beginning and end of the effective growing season.

The results of this study advance science by extending our understanding of the boundaries of the cultivatable area of *Miscanthus* to the North and East of geographical Europe. The wide diversity of germplasm from *M. sinensis, M. sacchariflorus* and several of their hybrids used in this experiment had informed breeders of the trait combinations needed to maximize biomass production in these regions with diverse environmental conditions.

## Author contributions

CN: corresponding author; AH: data analysis and manuscript editing; OK, MÖ, HS, IT, TV, AA, NK, JM, MM, and LT: data collection; YI: data analysis; AK: data analysis and manuscript editing; HM and KS: germplasm supply; IL: project leader; JC: project coordinator and manuscript editing.

### Conflict of interest statement

The authors declare that the research was conducted in the absence of any commercial or financial relationships that could be construed as a potential conflict of interest.
